# “Elastic stretch cavity building” system in endoscopic thyroidectomy *via* the axillary approach: a case series

**DOI:** 10.3389/fonc.2023.1167949

**Published:** 2023-04-27

**Authors:** Gaoxiang Chen, Xiaochun Ji, Hai Zhang, Yong Luo, Weizhu Wu, Weifeng Teng, Jianan Zhang, Shaocheng Zhou

**Affiliations:** Department of Thyroid and Breast Surgery, Ningbo Medical Center Lihuili Hospital, Ningbo, China

**Keywords:** endoscopic thyroidectomy, axillary approach, cavity builder, cosmetic satisfaction, elastic stretch cavity building, case series

## Abstract

**Background:**

Patients undergoing conventional endoscopic thyroidectomy *via* the axillary approach, which is commonly used clinically, suffered from a range of postoperative complications. This study aimed to prevent postoperative complications and evaluate patients’ satisfaction with cosmetic outcomes in endoscopic thyroidectomy *via* the axillary with the use of “Elastic Stretch Cavity Building” System.

**Methods:**

In this retrospective case series study, the clinical data of patients who were admitted to the Thyroid Surgery Department of Ningbo Medical Centre Lihuili Hospital between December 2020 and December 2021 for endoscopic thyroidectomy *via* the axillary approach under the “Elastic Stretch Cavity Building” System.

**Results:**

A total of 67 patients were included, all surgeries were successfully completed. The operation time was 75.61 ± 13.67 minutes; the postoperative drainage volume was 109.97 ± 37.54 ml; the average postoperative hospital stay was 4 (2-6) days. There was no skin ecchymosis, effusion or infection, hypocalcemia, convulsions, upper extremity dyskinesia, and temporary hoarseness after the surgery. The patients were satisfied with the cosmetic effects, and the cosmetic score was 4 (3-4).

**Conclusion:**

The “Elastic Stretch Cavity Building” System in endoscopic thyroid surgery *via* the axillary approach might reduce the risks of complications and achieve satisfactory results with the cosmetic outcomes.

## Introduction

Thyroid nodules, present in more than half of healthy individuals and most of which are asymptomatic, are 90% benign and 10% malignant (1, 2). Studies suggest that thyroid nodules may be related to thyroid dysfunction or local mass effect, and the current clinical problem is the identification and treatment of malignant lesions or lesions with a high risk of malignancy (3).

A total thyroidectomy is usually performed through an open transcervical incision, which leaves a noticeable scar in the anterior neck (4, 5). Endoscopic thyroidectomy, which has been gradually accepted due to its excellent cosmetic effects and its features of minimally invasive surgery (6, 7). With the development of endoscopic technology and the continuous adaptive improvement of surgical instruments, the approaches have gradually diversified (8, 9). Chung (Korea) et al. (10) first performed the full endoscopic thyroidectomy *via* the axillary approach with no inflation, and its largest advantage was that there were no scars on the skin of the neck and the anterior chest. As its operation approach was different from the traditional open surgery and the endoscopic thyroidectomy *via* the anterior chest, it provided another convenient approach for operator identification and the protection of the recurrent laryngeal nerve and parathyroid gland (11).

**Figure 1:**
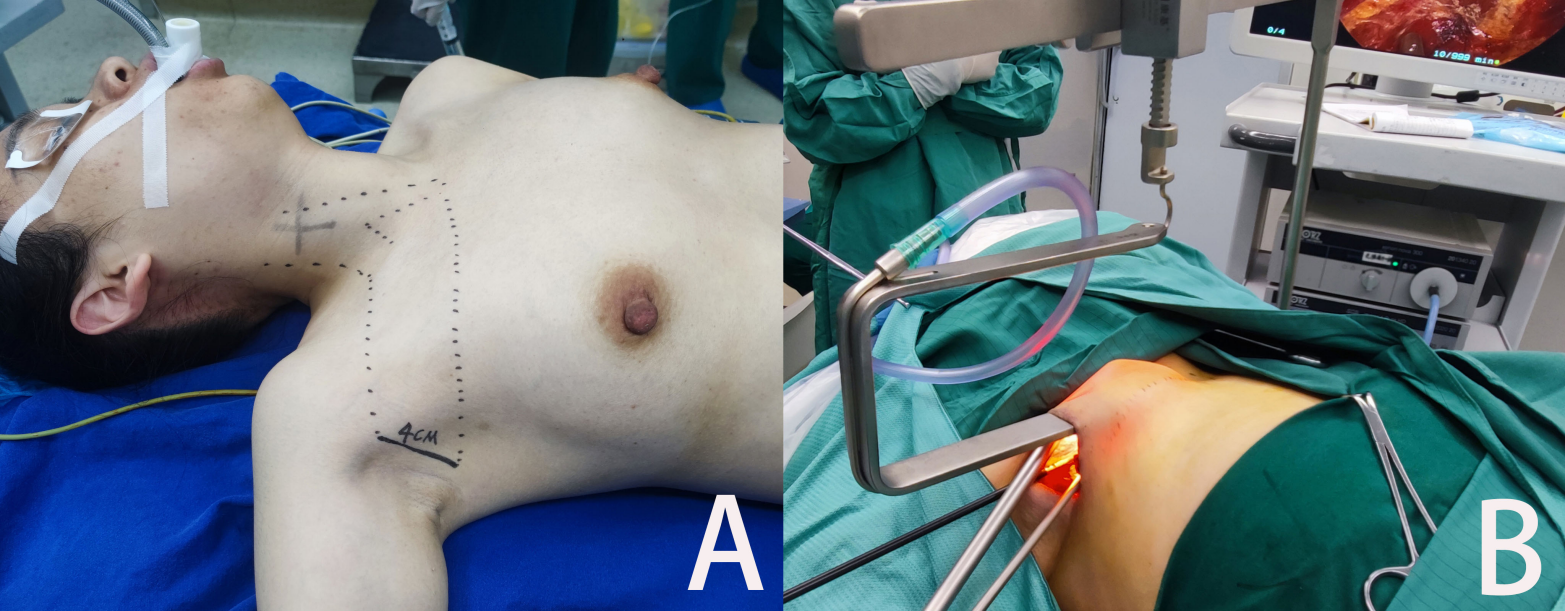
Endoscopic thyroidectomy through non-inflatable axillary approach **(A)** The patient’s position, incision location and the separation area of the anterior chest flap. **(B)** Surgical exterior view.

Currently, in the non-inflatable endoscopic thyroidectomy procedure commonly used in clinical practice *via* the axillary approach, a 4-5 cm axillary incision on the same side of the patient’s lesion is required, and a tunnel is created by separating a large flap in the anterior chest and rigidly lifting the flap in the anterior chest and neck muscles using a hook to create and maintain instrument channel and surgical operation space (**Figure 1**). Although this method can increase the volume of the working cavity to a certain extent and reduce the difficulty of surgery, it causes additional trauma to the patient (12). The authors’ team has carried out nearly 200 cases of non-inflatable endoscopic thyroidectomy. However, in the postoperative outpatient follow-ups, almost all patients suffered from varying degrees of skin numbness of the chest wall, sternocleidomastoid swelling on the surgery side, stiffness and other phenomena, and a few patients experienced shoulder pain and even movement dysfunction on the surgery side. These symptoms can last 3-6 months or even longer after surgery. It is urgent to consider and optimize alternative approaches to reduce the incidence, severity and duration of these complications.

The authors’ team has established a novel cavity building mode to shorten surgical incisions and improve the chest wall and neck postoperative discomfort. The natural position of the upper limbs in the supine position of the patient was used and the 1.5 cm incision made at the anterior axillary line along the skin patterns horizontally was used as the entrance of the observation lens. The auxiliary incisions for operation rods on both sides were located in the position where it could be easily covered by the underwear strap and on the upper lateral plica of the breast. The “Elastic Stretch Cavity Building” System which mainly used the mechanical cavity building mode supplemented by the inflation cavity building was formed in the operating space. This study aimed to explore the use of the “Elastic Stretch Cavity Building” System in endoscopic thyroid surgery *via* the axillary approach to prevent postoperative complications and to evaluate patients’ satisfaction with cosmetic outcomes.

## Methods

### Study design and participants

This retrospective case series study retrospective enrolled patients who underwent endoscopic thyroidectomy *via* the axillary approach under the “Elastic Stretch Cavity Building” System with the self-developed “elastic stretch cavity builder” at Thyroid Surgery Department of Ningbo Medical Centre Lihuili Hospital between December 2020 and December 2021. The work has been reported in line with the PROCESS (13).

Inclusion criteria: 1) Confirmed thyroid tumor and underwent endoscopic thyroidectomy, 2) The target lesions were confined to one lobe of the gland, the tumor did not invade the glandular envelope and surrounding tissues and organs, and no lymph node metastasis was indicated by preoperative imaging, 3) Tumors ≤ 2cm, with The Bethesda System for Reporting Thyroid Cytology (TBSRTC) classification of category 3 or higher, benign thyroid tumors ≤ 4.5cm (14). Exclusion criteria: 1) Periodical follow-up data were incomplete, 2) Concomitant secondary hyperthyroidism, 3) The patient suffered from other malignant diseases. This work has been carried out in accordance with the Declaration of Helsinki (2000) of the World Medical Association. The studies involving human participants were reviewed and approved by Ningbo Medical Center Lihuili Hospital (ethics approval number: DYLL2017009). Written informed consent for participation was not required for this study in accordance with the national legislation and the institutional requirements. Written informed consent was obtained from the individual(s) for the publication of any identifiable images or data included in this article.

### Typical surgical procedures and “elastic stretch cavity building” system

Except for cystic tumors, all patients underwent preoperative fine-needle aspiration biopsy and intraoperative rapid pathological section. Before surgery, cervical color Doppler ultrasonography and CT scan were performed to assess the location and size of the tumor.

### Equipment preparation

**Figure 2:**
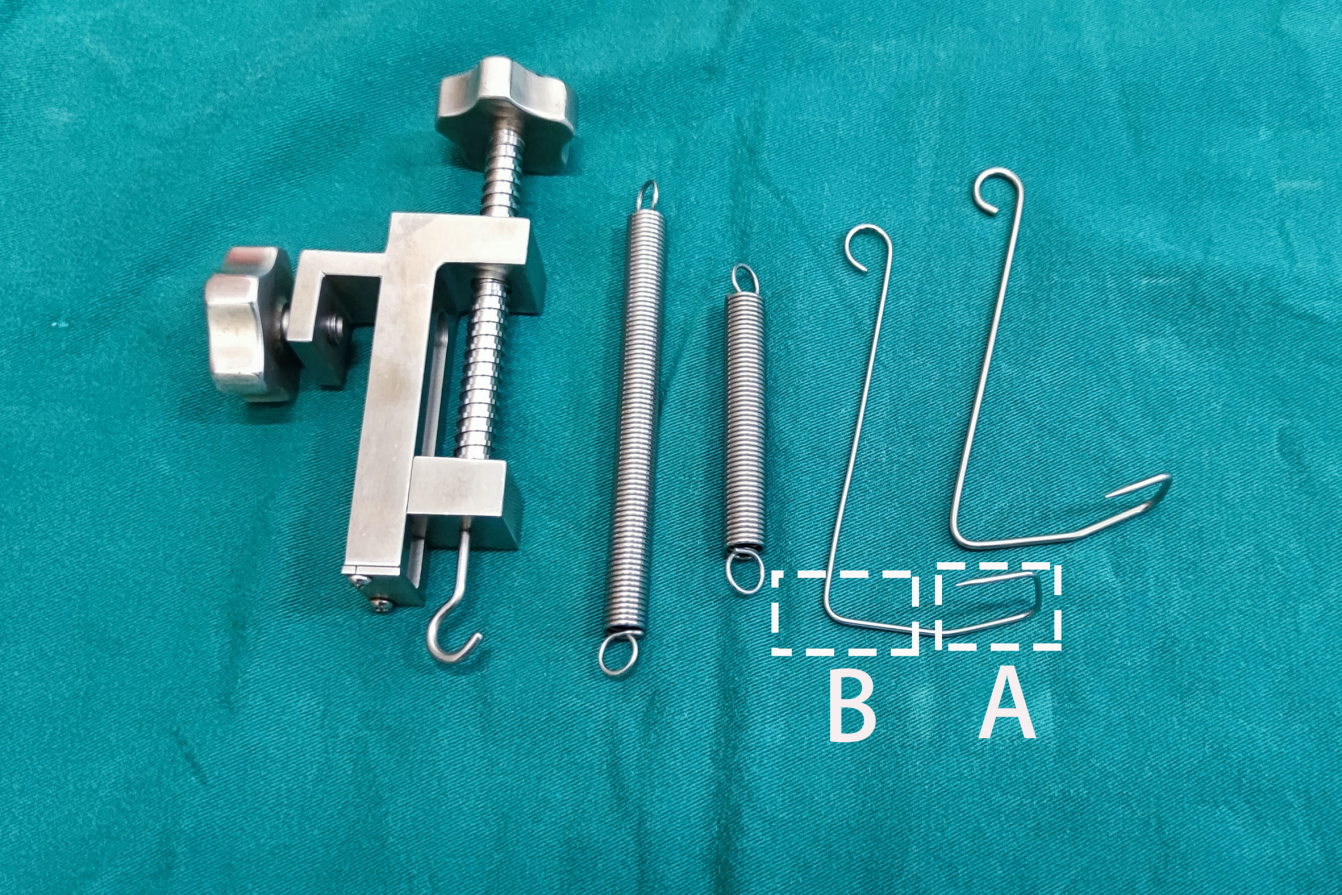
“Elastic Stretch Cavity Building” System (except for L-shaped brackets). Shows the adjuster, different types of springs, and different types of U-shaped hooks. **(A)** The first segment of the U-shaped hook; **(B)** The second segment of the U-shaped hook.

**Figure 3:**
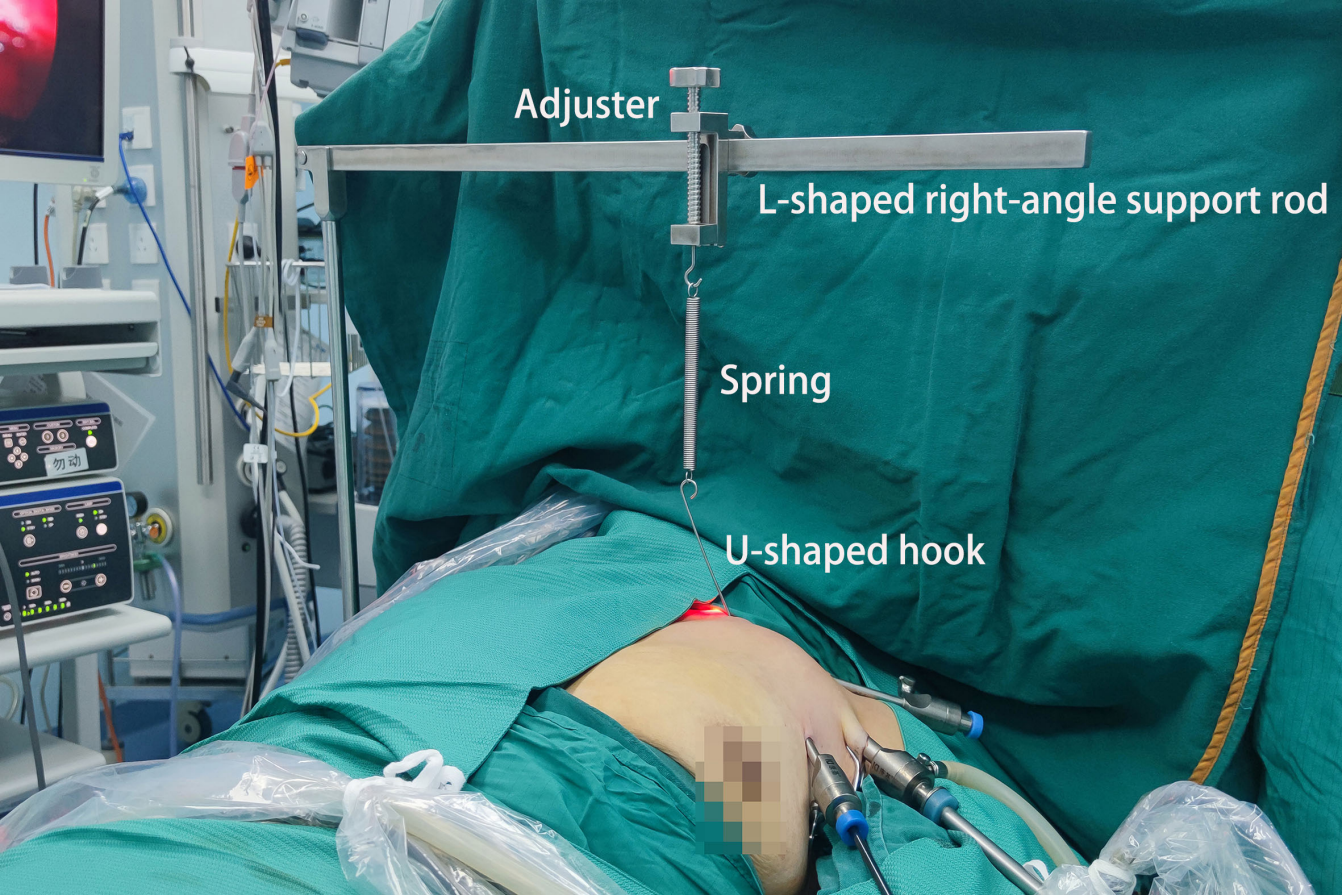
The usage scenario of the elastic stretch cavity builder.

Laparoscopic high-definition imaging system (OLYMPUS, STORZ), ultrasonic knife unit (Johnson & Johnson, USA). Common endoscopic operation instruments. The elastic stretch cavity builder: The L-shaped right-angle support rod (connected to the operating bed) The self-developed segmented U-shaped hook (1.8mm diameter, consisting of two segments, with a total length of 4 or 5cm). An adjuster which can generate a distance of 5cm up and down. Springs (two lengths: 5cm, 8cm. strength: 10N/cm) that connect the hook to the adjuster. (Patent No.: ZL 2015 2 0962366.7; ZL 2016 2 0599586.2; ZL 2019 2 0322833.8; ZL 2021 2 0444399.8) (**Figures 2**, **3**).

### Typical surgery procedures

Endotracheal intubation was used for general anesthesia. The patient’s head was tilted slightly to the healthy side and the neck was tilted back. The patient’s shoulder joint and upper limbs were in their natural positions when the patient was in the supine position, with no artificial abduction and rotation externally.

**Figure 4:**
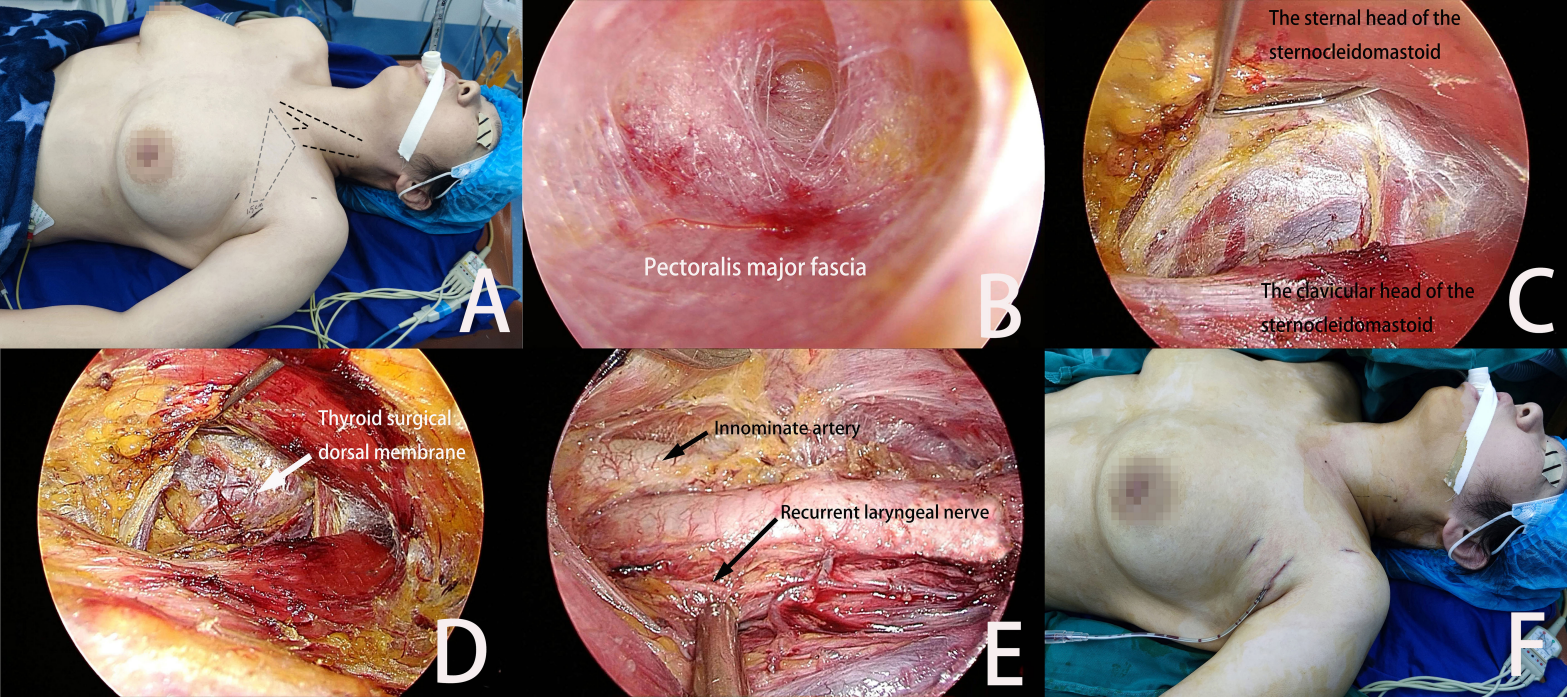
**(A)** The patient’s position and the incision location. The grey area is the separation area of the anterior chest flap. **(B)** The tunnel pre-separation method is used to establish the initial space in the anterior chest area. **(C)** The U-shaped hook lifts the sternal head of the sternocleidomastoid to establish an intermuscular channel. **(D)** The U-shaped hook lifts the neck muscles to create an operation space in the neck. **(E)** Postoperative view of lobectomy. **(F)** Postoperative drainage and the incision.

The ipsilateral axillary approach was adopted, and a 1.5 cm incision was made at the anterior axillary line along the skin patterns horizontally as the observation hole. A 0.5cm auxiliary incision was made at the intersection of the line connecting the observation hole and the ipsilateral nipple and the upper lateral plica of the breast. A 0.5cm auxiliary incision was made on the inside of the subclavian shoulder joint (this incision was located under the shoulder strap of the woman’s underwear) (**Figure 4A**).

10-20 ml of 1/200,000 epinephrine saline was injected to the surface of the pectoralis major fascia *via* infiltrating injection, and a flap separator was used for undermining dissection. A trocar was placed in the incision and CO_2_ was injected. The pressure was maintained at 6 mmHg. After the endoscope was entered, an ultrasonic knife or an electrocoagulation hook was used to separate the anterior chest flap inward and upward to the ipsilateral clavicle plane along the surface gap of the pectoralis major fascia (**Figure 4B**).

After the clavicle was passed, the lower 1/3 of the sternocleidomastoid was exposed and its sternal head and clavicular head were identified and separated. The U-shaped hook punctured through the skin flap and was placed into the operating cavity. The first segment of the hook was placed under the sternal head of the sternocleidomastoid. The hook was pulled upward vertically to connect to the adjuster through a spring. Then it was adjusted to an appropriate height (**Figure 4C**).

The separation was continued to the neck. The potential gap between the cervical sheath and the lateral edge of the ribbon-shaped muscle was opened and the second segment of the U-shaped hook was put into the surgical cavity. The anterior cervical ribbon-shaped muscle was lifted to expose the thyroid lobe on the surgery side. The anterior cervical operating space was formed, and its upper end reached the upper pole of the thyroid, its lower end reached the suprasternal fossa, and its inner side reached the thyroid isthmus (**Figure 4D**).

A continuous negative pressure suction tube was inserted into the surgical cavity through the observation hole and the inflation pressure of CO_2_ was adjusted to 3 mmHg. The continuous negative pressure suction in the cavity was combined with a high flow of CO_2_ (20L/min). The gland lobectomy was completed under this arrangement, and the surgical procedures were the same as those of the author’s previous reports (15) (**Figure 4E**).

All specimens were bagged, which were taken out through the observation hole and sent for frozen pathology. If the pathological results indicated malignancy, the ipsilateral central lymph node dissection would be performed. After the operation was completed, the surgical cavity was rinsed with distilled water. A high-vacuum negative pressure drainage tube was placed inside the cavity and it poked out through the wound near the main incision (**Figure 4F**). The above procedures of surgery conform to the routine surgical procedures of the department.

### Data collection and definition

Data including patient’s gender, age, time of surgery, postoperative drainage, postoperative hospital stay and pathological findings were collected. Postoperative complications were also collected during follow-up for at least 6 months. Postoperative complications included skin ecchymosis, effusion or infection, hypocalcemia, convulsions, upper extremity dyskinesia, and temporary hoarseness. Cosmetic satisfaction scores were performed three months after surgery (1: very dissatisfied; 2: dissatisfied; 3: satisfied; 4: very satisfied).

### Statistical analysis

Only descriptive analysis was performed in this study.

## Results

A total of 67 patients were included in this study, 13 (19.40%) of them were men and 54 (80.60%) were women, with an average age of 42.53 ± 10.63 years. Among them, 59 patients (88.06%) had intraoperative pathological findings suggestive of papillary carcinoma and underwent unilateral thyroid lobectomy and thyroid isthmus resection with lymph node dissection in the central region, 8 patients (11.94%) had intraoperative pathology suggestive of nodular goiter and underwent unilateral subtotal or near-total thyroidectomy. The operation time was 75.61 ± 13.67 minutes, the postoperative drainage volume was 109.97 ± 37.54 ml and the average postoperative hospital stay was 4 (2-6) days. No significant recurrence was seen on ultrasound re-examination at 6 to 18 months of postoperative follow-up. There was no skin ecchymosis, effusion or infection, hypocalcemia, convulsions, upper extremity dyskinesia, and temporary hoarseness after the surgery. The patients were satisfied with the cosmetic effects with cosmetic score of 4 (3-4) (**Table 1**).

**Table 1:** Basic information of patients.

Characteristic	Value
Gender	
Male, N (%)	13 (19.40)
Female, N (%)	54 (80.60)
Age (year), mean ± SD	42.53 ± 10.63
Intraoperative pathological findings	
Papillary carcinoma, N (%)	59 (88.06)
Nodular goiter, N (%)	8 (11.94)
Operation time, min, mean ± SD	75.61 ± 13.67
Postoperative drainage volume, ml, mean ± SD	109.97 ± 37.54
Postoperative hospital stay, day, median(range)	4 (2-6)
Follow-up time, month, median(range)	11(6-18)
Cosmetic score, median(range)	4 (3-4)

## Discussion

Endoscopic thyroidectomy using an “Elastic Stretch Cavity Building” System *via* axillary approach showed that surgeries in all cases were successful and achieved satisfactory results with the cosmetic outcomes.

After trying different body positions in the early stage (4–6), the authors’ team has found that abducting the patient’s upper limbs to 90° or a larger angle may cause shoulder joint pain and even upper limb movement dysfunction after the surgery. Therefore, the natural position of the upper limbs in the supine position of the patient was used. The 1.5 cm incision made at the anterior axillary line along the skin patterns horizontally was used as the entrance of the observation lens. With this more appropriate approach than the breast approach (16), postoperative upper limb dysfunction can be avoided as much as possible, ensuring cosmetic results and surgical convenience. The auxiliary incisions for operation rods on both sides were located in the position where it could be easily covered by the underwear strap and on the upper lateral plica of the breast. The three independent incisions could separate the channel of the endoscope from that of the operating instruments, which alleviated the interference between the endoscope and the instruments and the chopstick effect of the operating rods caused by the original single incision in the axilla (16, 17). Therefore, the surgical difficulty was reduced and the surgical safety was improved. In the meantime, the minimally invasive features and cosmetic effects were guaranteed to the greatest extent.

As the surgery is mainly performed in the neck, the establishment and maintenance of the neck operation space are necessary (18, 19). The separation area of the skin flap should be as small as possible in principle on the premise that it is sufficient for the operation (12). In the process of establishing the initial space in the anterior chest area, the tunnel pre-separation method similar to the cavity building mode *via* the thoracic breast approach was adopted. The non-inflatable axillary approach we used in the past required a larger(4-5cm) skin incision and extensive skin flap separation in the anterior chest area, but the new system avoided the long, large anterior chest flap separation from the axilla to the neck. At the same time, the non-inflatable axillary approach required the placement of hard hooks under the skin flap and muscles, the area where the hooks were pulled was the area where the supraclavicular nerves are distributed. Therefore, the postoperative numbness and discomfort in the anterior chest area were more pronounced. The new system directly pulls the neck muscles, avoiding pulling the chest flap distributed by the supraclavicular nerve. Therefore, in addition to reduced cavity building time, the separation area of the anterior chest flap was also decreased, alleviating the trauma and the postoperative discomfort in the anterior chest area caused by skin flap separation. The spring component added in the cavity builder can control the lifting tension of the hook, avoiding the secondary injury of the anterior chest flap and neck muscles due to excessive tension, which allows for rapid recovery in minimally invasive surgery.

Endoscopic surgery needs a stable and sufficient operating space (20, 21). The initial space was made by separating the anterior chest area. As the space was relatively small, the conventional CO_2_ inflatable cavity building (6 mmHg) was adopted to uniformly expand the loose connective tissue space on the surface of the pectoralis major fascia (8, 22–24). Thus, the process of flap separation would be simple and smooth. The natural space in the muscle belly of the sternocleidomastoid was used as a channel, which went from the anterior chest space to the neck space with the clavicle as the boundary. After the space of the two ends of the sternocleidomastoid was identified and separated, it was difficult to continue to provide a stable and sufficient operating space by CO_2_ inflation only. Therefore, the self-developed segmented U-shaped hook was used to vertically lift the muscle belly of the sternal head to establish a stable intermuscular channel (17). The potential space between the lateral edge of the ribbon-shaped muscle and the cervical sheath was found and separated. The segmented U-shaped hook was put in the space between the gland and the ribbon-shaped muscle. The hook lifted most of the gland to the opposite upper side to establish the operating space in the neck. The “Elastic Stretch Cavity Building” System which mainly used the mechanical cavity building mode supplemented by the inflation cavity building was formed in the operating space. At this time, CO_2_ perfusion was no longer necessary for maintaining the space. The gas leak during the operation would not cause surgery interruption, and there was a stable and continuous observation field during the whole operation process. The combination of the cavity builder which lifted the neck muscles, the hybrid cavity building mode with low pressure and high flow, and the continuous negative pressure suction could generate continuous gas exchange in the operating cavity, thereby quickly taking away the smoke and keeping the vision field clear.

A limitation of this study was that it was a retrospective study, a future randomized controlled trial could address this limitation. In addition, novel cavity building mode certainly has its shortcomings, compared with the non-inflatable cavity building mode. Although the high-flow gas exchange can take away most of the smoke, the mist generated by the operation in the depth of the cavity space inevitably blurs the lens as the surgery progresses, thus affecting the smoothness of the operation. In addition, since the end width of the hook is relatively narrow, the cavity gap generated by lifting the hook is also relatively small, bringing certain difficulties to the operation. Subsequent increases in random sample size are necessary to avoid selection bias and causality bias.

## Conclusions

In summary, the “Elastic Stretch Cavity Building” System in endoscopic thyroid surgery *via* the axillary approach might reduce the risks of complications. More prospective studies are needed to confirm the findings of the present study.

## Data availability statement

The original contributions presented in the study are included in the article/**Supplementary material**. Further inquiries can be directed to the corresponding author.

## Ethics statement

This work has been carried out in accordance with the Declaration of Helsinki (2000) of the World Medical Association. The studies involving human participants were reviewed and approved by Ningbo Medical Center Lihuili Hospital (ethics approval number: DYLL2017009). Written informed consent for participation was not required for this study in accordance with the national legislation and the institutional requirements. Written informed consent was obtained from the individual(s) for the publication of any identifiable images or data included in this article.

## Author contributions

GC provided the conceptualization of this study, drafted the manuscript, and performed data analysis. XJ, HZ contributed to the study’s design and collection of data. WT, JZ and SZ worked on investigation and data collection. YL and WW conducted the critical revision of the manuscript. All authors contributed to the article and approved the submitted version.
